# Lung Ultrasound in Cystic Fibrosis: A Systematic Review of Clinical Utility, Scoring Systems, and Correlation With Structural and Functional Outcomes

**DOI:** 10.7759/cureus.104775

**Published:** 2026-03-06

**Authors:** Diana Popin, Mihaela Dediu, Alexandru Dabica, Iulia Ioan, Ioana M Ciuca

**Affiliations:** 1 Pediatric Department, Doctoral School, “Victor Babes” University of Medicine and Pharmacy, Timisoara, ROU; 2 Pediatric Department, “Victor Babes” University of Medicine and Pharmacy, Timisoara, ROU; 3 Obstetrics and Gynaecology Department, Doctoral School, “Victor Babes” University of Medicine and Pharmacy, Timisoara, ROU; 4 Pediatric Functional Explorations Department, Children’s Hospital, Nancy University Hospital Center, Nancy, FRA; 5 Development-Adaptation-Handicap (DevAH) Research Unit, Faculty of Medicine, University of Lorraine, Nancy, FRA

**Keywords:** cystic fibrosis, imaging, lung ultrasound, lung ultrasound score, structural lung disease, thoracic ultrasound

## Abstract

Background lung ultrasound (LUS) has emerged as a potential radiation-free imaging modality for the evaluation of cystic fibrosis (CF) lung disease. However, its clinical utility, the role of CF-specific LUS scoring systems, and correlations with structural and functional outcomes remain incompletely defined. The objective of this review was to systematically evaluate the role of LUS in cystic fibrosis, with particular focus on CF-specific scoring systems, diagnostic performance, and correlations with imaging and functional disease severity.

A systematic search of PubMed and Web of Science was conducted for studies published between January 2016 and January 2026 evaluating LUS in individuals with cystic fibrosis. Prospective and retrospective observational studies assessing LUS findings, scoring systems, or clinical outcomes were included. Screening, data extraction, and risk-of-bias assessment were performed independently by two reviewers in accordance with Preferred Reporting Items for Systematic Reviews and Meta-Analyses (PRISMA) 2020 recommendations. Due to heterogeneity in study design, ultrasound protocols, and outcomes, results were synthesized qualitatively.

Nine studies involving 390 individuals with cystic fibrosis met the inclusion criteria, with most cohorts consisting predominantly of pediatric patients. Four studies developed CF-specific LUS scoring systems, while others applied existing or non-CF-specific scores or descriptive protocols. LUS consistently detected peripheral abnormalities, such as subpleural consolidations, pleural irregularities, and interstitial patterns, and frequently identified lesions not visible on chest radiography. CF-specific LUS scores demonstrated strong correlations with computed tomography (CT) structural severity, spirometry, and lung clearance index (LCI), particularly in patients with moderate-to-advanced disease. Correlations were weaker in cohorts with predominantly mild disease or when non-CF-specific scoring systems were used. LUS showed limited sensitivity for central airway pathology, including bronchiectasis and mucus plugging.

LUS is a feasible, radiation-free imaging modality capable of detecting and monitoring peripheral lung disease in cystic fibrosis, particularly when CF-specific scoring systems are applied. While it cannot replace CT for comprehensive structural evaluation, LUS may serve as a valuable complementary tool for longitudinal monitoring. Further standardization and multicentre longitudinal studies are required to define its role in routine CF care.

## Introduction and background

Cystic fibrosis (CF) is one of the most common life-limiting autosomal recessive disorders among the Caucasian population. It is caused by mutations in the cystic fibrosis transmembrane conductance regulator (CFTR) gene, leading to defective chloride and bicarbonate transport across epithelial surfaces [1]. Impaired mucociliary clearance and chronic airway inflammation result in progressive structural lung damage, including bronchiectasis, mucus plugging, atelectasis, and air trapping, which remain the major determinants of morbidity and mortality in people with CF (pwCF) [1].

Over the past decade, the natural history of cystic fibrosis has evolved with the introduction of highly effective CFTR modulator therapies and advances in multidisciplinary care. These developments have improved survival and quality of life, shifting clinical priorities toward earlier detection and longitudinal monitoring of structural lung disease [2]. In this context, the need for radiation-sparing imaging strategies has become increasingly relevant.

High-resolution computed tomography (HRCT) remains the reference standard for assessing structural lung abnormalities in CF due to its high sensitivity for bronchiectasis, air trapping, and parenchymal changes [2]. However, concerns related to cumulative ionizing radiation exposure, particularly in children and young adults requiring lifelong surveillance, as well as cost and the need for sedation in younger patients, have driven interest in alternative imaging modalities [3]. Magnetic resonance imaging (MRI) offers a radiation-free alternative and has gained increasing acceptance, with CF-specific MRI protocols undergoing substantial validation in recent years [2,3]. In current clinical practice, structural lung disease in cystic fibrosis is primarily monitored using periodic high-resolution computed tomography (HRCT), while magnetic resonance imaging is increasingly adopted in specialized centers as a radiation-free alternative, particularly in pediatric populations. Despite these advances, the need for accessible imaging tools that can be used repeatedly without radiation exposure continues to drive interest in complementary bedside modalities.

Lung ultrasound (LUS) has emerged as a non-invasive, bedside imaging technique capable of detecting peripheral lung abnormalities such as consolidations, pleural irregularities, and interstitial patterns. LUS does not directly visualize aerated lung parenchyma but relies on the interpretation of characteristic artifacts generated at the pleural interface. Because ultrasound waves are reflected by air, assessment is largely limited to peripheral lung structures, while deeper central airway pathology remains less accessible. This characteristic is particularly relevant in cystic fibrosis, where many structural abnormalities extend to the pleural surface and can therefore be detected using ultrasound. Its absence of ionizing radiation, portability, and repeatability make it particularly attractive for longitudinal monitoring in cystic fibrosis [4]. LUS is already widely used in pediatric and adult respiratory medicine for the assessment of pneumonia, pleural disease, and interstitial syndromes, and its application in CF has gained growing interest in recent years [5].

Recent studies have explored the use of LUS in CF for detecting structural abnormalities, monitoring disease progression, and evaluating treatment response. Several CF-specific LUS scoring systems have been proposed, with varying degrees of correlation with computed tomography findings, pulmonary function, and microbiological status [6]. A recent narrative review by Boni et al. summarized emerging evidence on the role of LUS in cystic fibrosis and highlighted its potential as a complementary imaging modality while emphasizing the need for further validation and standardization [6].

Despite growing interest in LUS for cystic fibrosis, important uncertainties remain regarding the comparative performance of different LUS scoring systems, their correlation with structural and functional disease severity, and their potential clinical role in routine monitoring. The increasing life expectancy of people with CF and the need for repeated, radiation-sparing imaging further underscore the relevance of clarifying the utility of LUS in this setting [7].

This systematic review aims to evaluate the current evidence regarding the role of LUS in cystic fibrosis, with particular focus on CF-specific scoring systems, correlations with computed tomography and chest radiography, associations with pulmonary function and microbiological status, and potential clinical applications in disease monitoring.

## Review

Materials and methods

Protocol and Registration

This review was not preregistered. Formal PROSPERO registration was not pursued, given the diagnostic-methodological nature of the topic, which integrates clinical utility, scoring approaches, and correlations with structural and functional outcomes; this scope is not compatible with PROSPERO eligibility criteria for health outcome-focused systematic reviews and anticipates a predominantly narrative synthesis. The protocol was defined a priori, no major deviations occurred during study conduct, and the review was performed in accordance with Preferred Reporting Items for Systematic Reviews and Meta-Analyses (PRISMA) 2020 recommendations. Due to the niche nature of the subject and the paucity of pediatric-only evidence, adult cohorts were retained to maximize data availability and ensure a comprehensive synthesis. Two reviewers underwent calibration prior to independent screening, with disagreements resolved by consensus. Screening and workflow management were conducted using the Rayyan platform. Ethical approval was not required, as all data originated from published studies.

Literature Search Strategy

A systematic search was conducted in PubMed and Web of Science covering the period January 2016 to January 2026. Controlled vocabulary and free-text terms relating to cystic fibrosis, LUS, and thoracic ultrasound imaging were used. The PubMed strategy combined MeSH headings for cystic fibrosis and ultrasonography with text words for lung and thoracic ultrasound, and equivalent syntax was applied to the other databases. Backward citation chaining of all included full texts and relevant reviews was performed to identify additional studies. No restrictions on study design, publication type, or language were applied at the search stage. Duplicate records were removed prior to screening.

Eligibility Criteria and Study Selection

The review included prospective and retrospective observational studies, cross-sectional studies, and clinical trials evaluating LUS in individuals of any age with cystic fibrosis. Studies were required to report at least one clinical, diagnostic, or monitoring outcome related to LUS. Only full-text, peer-reviewed articles published in English were considered.

The review excluded case reports, case series, conference abstracts, conference proceedings, posters, editorials, letters, commentaries, theses, animal or in vitro studies, and studies not involving LUS or cystic fibrosis. Narrative and systematic reviews were not included in the synthesis but were screened for relevant citations. The predefined inclusion and exclusion criteria are summarized in Table 1.

**Table 1:** Inclusion and exclusion criteria

Inclusion Criteria	Exclusion Criteria
Prospective and retrospective observational studies evaluating lung ultrasound in cystic fibrosis	Case reports and case series
Cross-sectional studies and clinical trials	Conference abstracts, proceedings, and posters
Participants of any age with cystic fibrosis	Editorials, letters, and commentaries
Studies reporting clinical, diagnostic, or monitoring outcomes of lung ultrasound	Theses and gray literature
Full-text, peer-reviewed articles	Animal or in vitro studies
Articles published in English	Studies not involving lung ultrasound or cystic fibrosis
—	Narrative and systematic reviews (screened only for references)

Data Extraction and Risk-of-Bias Assessment

Two reviewers independently extracted data using a predefined data extraction form. Extracted variables included study characteristics (year of publication, country, study design, and population), participant characteristics (age group and sample size), LUS protocol details (scanning approach, scoring systems, and assessed lung findings), comparator imaging modalities when applicable (chest radiography or computed tomography), and reported outcomes related to diagnostic performance, disease severity, or clinical utility. Discrepancies were resolved by consensus. Study authors were contacted when clarification or additional data were required.

Risk of bias was assessed independently by two reviewers using the QUADAS-2 tool for diagnostic accuracy studies. The assessment covered four domains: patient selection, index test, reference standard, and flow and timing. Overall risk-of-bias judgments were summarized descriptively. Given the limited number of included studies (<10), a formal assessment of publication bias was not undertaken.

Data Synthesis

Due to substantial heterogeneity in study design, LUS protocols, scoring systems, reference standards, and reported outcomes, quantitative pooling of results was not feasible. Therefore, findings were synthesized qualitatively using a narrative approach. Studies were grouped and summarized according to population characteristics (pediatric, adult, or mixed cohorts), clinical setting, LUS methodology, and reported outcomes, including diagnostic performance, correlation with reference imaging modalities, and assessment of disease severity or clinical status. Where applicable, trends and consistencies across studies were highlighted.

Results

Study Selection

**Figure 1:**
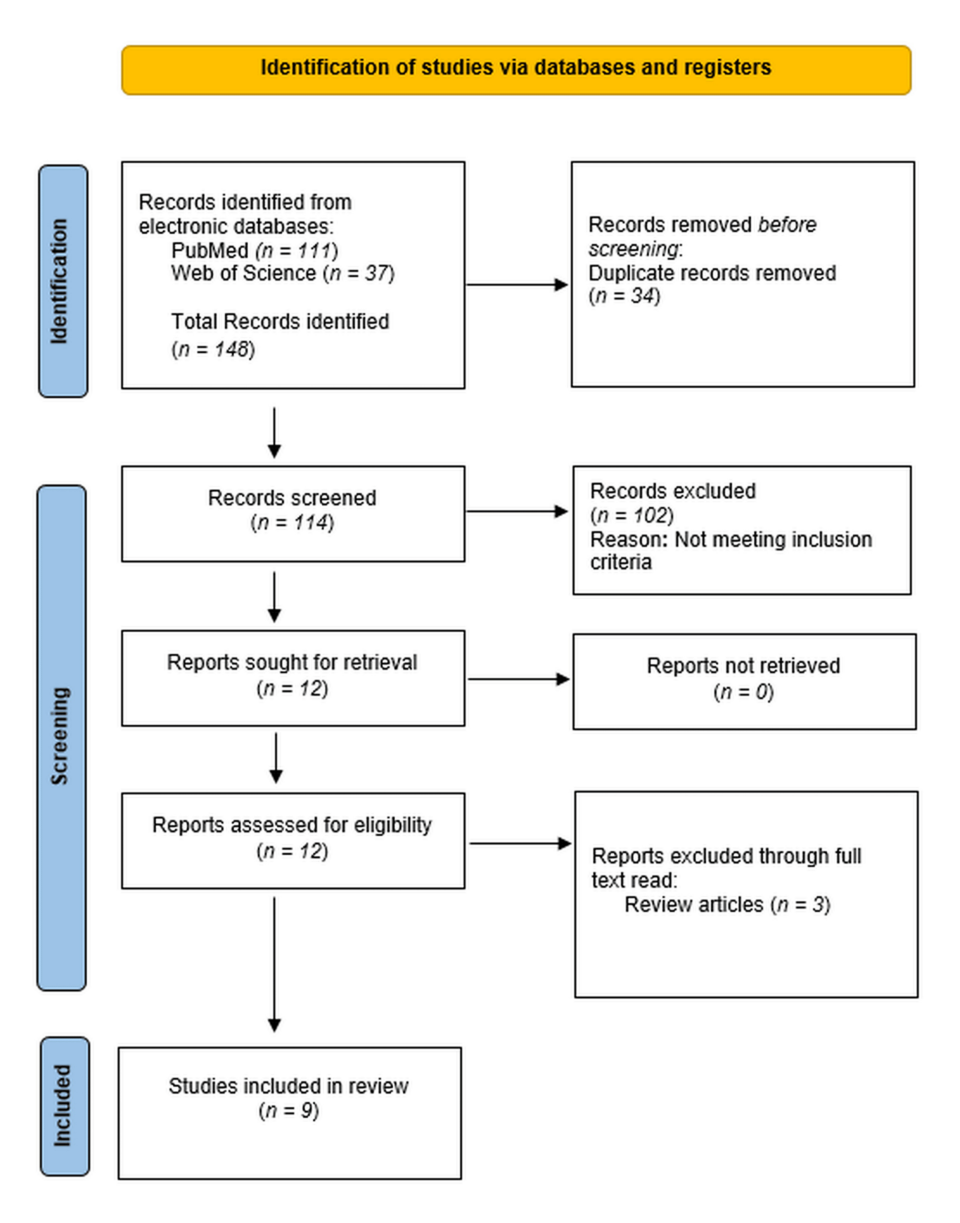
PRISMA 2020 flow diagram of study selection PRISMA: Preferred Reporting Items for Systematic Reviews and Meta-Analyses

The database search identified 148 records. After removal of 34 duplicate records, 114 records were screened based on title and abstract, of which 102 were excluded for not meeting the inclusion criteria. Twelve full-text articles were assessed for eligibility, and three were excluded because they were review articles. Nine studies met the inclusion criteria and were included in the qualitative synthesis. The study selection process is illustrated in the PRISMA 2020 flow diagram (Figure 1).

Study Characteristics

The nine included studies were published between 2019 and 2025 and evaluated LUS in pediatric, adult, or mixed-age populations with cystic fibrosis across outpatient, inpatient, and specialized care settings. Most studies focused predominantly on pediatric cohorts, while a minority included adolescents and adults. Study designs comprised prospective cross-sectional studies, pilot feasibility studies, and observational cohort analyses conducted in cystic fibrosis referral centers.

Sample sizes varied across studies, reflecting the exploratory nature of LUS application in cystic fibrosis. For descriptive purposes, cohorts were classified as small (<30 participants), moderate (30-100 participants), or large (>100 participants). LUS protocols differed with respect to scanning technique, number of lung zones assessed, and scoring approaches. Commonly evaluated ultrasound findings included B-lines, subpleural consolidations, pleural line irregularities, and regional aeration loss. Reference standards most frequently consisted of chest computed tomography or chest radiography, while several studies also explored correlations with pulmonary function tests, clinical status, or disease severity scores. Key study characteristics are summarized in Table 2.

**Table 2:** Characteristics of studies evaluating lung ultrasound in cystic fibrosis Abbreviations: CF: cystic fibrosis; LUS: lung ultrasound; CXR: chest radiography; CT: computed tomography † Cohort size classification: Small (<30 participants); Moderate (30–100 participants); Large (>100 participants)

Study (First Author, Year)	Country	Study Design	Population	Age Group	Cohort Size†	Clinical Setting	LUS Application	Reference Standard
Strzelczuk-Judek et al., 2019 [8].	Poland	Pilot observational study	CF	Pediatric	Small	Specialized CF center	Diagnostic evaluation	CXR
Peixoto et al., 2020 [9].	Portugal	Observational study	CF	Mixed	Moderate	Hospital setting	Structural lung disease evaluation	Chest CT
Hassanzad et al., 2021 [10].	Iran	Prospective observational	CF	Mixed	Moderate	Hospital setting	Exacerbation detection	CXR
Ciuca et al., 2022 [11].	Romania	Feasibility study	CF	Pediatric	Small	Tertiary care center	Comparison with CT	Chest CT
Jaworska et al., 2023 [12].	Poland	Prospective cross-sectional study	CF	Pediatric	Large	Outpatient CF clinic	Disease severity assessment	Chest CT
Curatola et al., 2024 [13].	Italy	Observational cohort study	CF	Mixed	Moderate	Outpatient pulmonology clinic	Routine clinical assessment	Chest CT / clinical evaluation
Marzook et al., 2024 [14].	Multicenter	Prospective cross-sectional study	CF ± PCD	Pediatric	Moderate	Specialized pediatric centers	Comparative diagnostic evaluation	CXR
Crispino et al., 2024 [15].	Italy	Prospective pilot case-control study	CF	Pediatric (preschool)	Small	CF referral center	Point-of-care LUS	-
Gräger et al., 2025 [16].	Germany	Prospective cross-sectional case–control study	CF	Pediatric	Small	CF specialty clinic	Diagnostic feasibility	-

Across the 9 included studies, LUS was performed in a total of 390 CF individuals, with study populations being predominantly pediatric [8-16]. Sample sizes varied substantially, ranging from small pilot cohorts of 17-18 participants to larger observational studies enrolling up to 131 patients, reflecting the exploratory and heterogeneous application of LUS in cystic fibrosis across different clinical settings.

Age was reported in all studies, although formats differed, with some authors providing mean values and others reporting medians or age ranges. Most cohorts consisted of children and adolescents, including preschool-aged populations, with reported ages spanning from 5 weeks to 18 years. Adult patients were included in a limited number of studies, primarily within mixed cohorts, with mean ages extending into young adulthood in those populations.

Genotype information was reported in several studies, though not uniformly. Among cohorts providing genetic data, the F508del mutation was the most frequently observed CFTR variant, present in both homozygous and compound heterozygous forms, while non-F508del variants were less commonly represented. However, genotype reporting was heterogeneous, precluding comprehensive pooled genetic analysis.

Data on airway microbiology were variably reported. When specified, *Pseudomonas aeruginosa* was the most frequently documented pathogen, followed by *Staphylococcus aureus*, with other organisms reported less consistently. Definitions and reporting of chronic infection differed across studies.

Given the heterogeneous and incomplete reporting of demographic, genetic, nutritional, and microbiological variables, quantitative pooling of patient-level characteristics was not appropriate. Reported clinical characteristics are therefore summarized descriptively and presented where available in Table 3.

**Table 3:** Key demographic and clinical characteristics of cystic fibrosis populations included in lung ultrasound studies Abbreviations: CF = cystic fibrosis; BMI = body mass index *Jaworska et al. reported microbiological status in all patients but did not provide a single pooled *Pseudomonas aeruginosa* percentage suitable for tabulation.

Study (Year)	CF patients (n)	Age (years)	Nutritional status (BMI / z-score)	Key genotype finding	Pseudomonas aeruginosa
Strzelczuk-Judek et al., 2019 [8].	48	Mean 11.9 ± 3.9	Mean BMI 16.7 ± 2.9	F508del homozygous 43.8%	43.8%
Peixoto et al., 2020 [9].	18	Mean 15.3 ± 4.4	Mean BMI 17.9 ± 2.9	F508del homozygous 38.9%	44.4%
Hassanzad et al., 2021 [10].	30	Mean 19.6 ± 5.5	BMI <5th centile: 66.7% (<20 y)	Not reported	Not reported
Ciuca et al., 2022 [11].	57	Mean 11.8 ± 5.5	Not reported	F508del homozygous 49.1%	33.3%
Jaworska et al., 2023 [12].	131	Median 7.1 (5 w–18 y)	Not reported	All genetically confirmed CF	Not reported*
Crispino et al., 2024 [15].	17	Mean 3.1 ± 2.1	Not reported	CF-causing variants predominant	Not reported
Curatola et al., 2024 [13].	29	Mean 27.1 ± 9.1	Mean BMI 21.5 ± 2.8	F508del homozygous 34.5%	51.7%
Marzook et al., 2024 [14].	30	Median 9.1 (IQR 6.9–12.2)	BMI z-score −0.23	F508del homozygous 53%	73%
Gräger et al., 2025 [16].	30	6–18 y	Mean BMI 18.1 ± 2.9	F508del homozygous 46.7%	Not reported

Risk-of-Bias Assessment Across Studies

Risk of bias was evaluated using the QUADAS-2 tool across four domains: patient selection, index test, reference standard, flow, and timing. Two studies were judged to be at an overall low risk of bias across all domains, while the remaining studies were rated as having low-moderate to moderate risk of bias. The most frequent sources of concern related to patient selection include non-consecutive or convenience sampling and small pilot cohorts, as well as unclear reporting regarding blinding between LUS and reference standard interpretation. The reference standards were generally appropriate and most commonly consisted of chest radiography or computed tomography. No study was excluded on the basis of risk-of-bias assessment. The detailed risk-of-bias assessment for each included study is summarized in Table 4.

**Table 4:** Risk of bias assessment using QUADAS-2 * Risk of bias was assessed using the QUADAS-2 tool across four domains: patient selection, index test, reference standard, and flow and timing. Overall risk of bias reflects the highest risk judgment across domains. “Unclear” indicates insufficient reporting rather than confirmed methodological flaws. Abbreviations: LUS = lung ultrasound

Study (Author, Year)	Patient Selection	Index Test (LUS)	Reference Standard	Flow & Timing	Overall Risk of Bias
Hassanzad, 2021 [10].	Low	Low	Low	Low	Low
Jaworska, 2023 [12].	Low	Low	Low	Low	Low
Curatola, 2024 [13].	Low	Low	Unclear	Low	Low–Moderate
Strzelczuk-Judek, 2019 [8].	Unclear	Unclear	Low	Unclear	Moderate
Peixoto, 2020 [9].	Unclear	Unclear (no blinding)	Low	Unclear	Moderate
Ciuca, 2022 [11].	Unclear	Low	Low	Unclear	Moderate
Marzook, 2024 [14].	High (convenience sampling)	Low	Unclear	Unclear	Moderate
Crispino, 2024 [15].	High (pilot study, small sample)	Low	Unclear	Unclear	Moderate
Gräger, 2025 [16].	Unclear	Low	Unclear	Unclear	Moderate

LUS Protocols and Reported Sonographic Findings

Across the nine included studies, LUS protocols and reported sonographic findings were heterogeneous and are summarized below [8-16].

Ultrasound probes and examination protocols: All included studies reported the use of different ultrasound probes and frequency ranges for LUS examinations [8-16]. High-frequency linear transducers (typically 5-12 MHz or 7-12 MHz) were most frequently used, particularly in pediatric populations [8,9,11-13]. Several authors additionally reported the use of microconvex (5-8 MHz) or convex probes (3-5 MHz), either alone or in combination with linear probes, particularly in older children, adolescents, or when deeper parenchymal assessment was required [9,10,14-16].

Scanning protocols varied between studies. Most authors reported a multi-zone scanning approach, including anterior and lateral lung regions, with posterior lung fields assessed when patient cooperation and clinical setting allowed [8,11,12]. No standardized scanning protocol was uniformly applied across studies.

LUS artifacts assessed in cystic fibrosis: Across studies, LUS was used to assess a range of sonographic artifacts and pathological findings associated with cystic fibrosis lung disease [8-16]. Reported findings included vertical artifacts (B-lines and related variants), subpleural and larger consolidations, atelectasis, pleural line abnormalities, and pleural effusions.

Reported normal LUS patterns: Normal LUS appearance was described by authors as the presence of lung sliding, a continuous pleural line, and horizontal A-lines, interpreted as markers of preserved lung aeration [9,12]. The absence or reduction of A-lines was reported as suggestive of underlying loss of aeration [12]. Z-lines were described as short vertical artifacts arising from the pleural line without erasing A-lines and were observed in both cystic fibrosis patients and healthy controls [12]. B-lines were the most consistently reported LUS artifact across the included studies [8-16]. Authors described B-lines as vertical hyperechoic artifacts extending from the pleural line to the bottom of the screen, moving synchronously with lung sliding and erasing horizontal A-lines. B-lines were reported as either isolated/focal or multiple and coalescent, forming interstitial patterns (“lung rockets”) [9,11,14]. The reported prevalence of interstitial-alveolar patterns varied substantially across studies, ranging from approximately 22% in stable cohorts to over 70% in patients with more advanced disease or during pulmonary exacerbations [8,10,15].

Jaworska et al. reported additional vertical artifacts, including I-lines and Am-lines, which were observed significantly more frequently in cystic fibrosis populations than in healthy controls [12].

Consolidations: Pulmonary consolidations were reported as hypoechoic subpleural lesions, sometimes associated with air bronchograms [8-16]. The prevalence of consolidations ranged from less than 10% in stable pediatric cohorts to over 60% in patients evaluated during pulmonary exacerbations [10,13]. Small subpleural consolidations were frequently reported [11,14].

Several studies reported good diagnostic performance of LUS for consolidation detection compared with computed tomography, with sensitivities exceeding 90% and specificities between 90% and 94% [10,11,13,15]. Moderate-to-strong correlations between LUS and CT findings were reported (r ≈ 0.77-0.79) [8,13].

Atelectasis: Atelectasis was reported as a form of subpleural consolidation, characterized by the absence of a dynamic air bronchogram and the presence of internal horizontal air artifacts [10,11]. LUS was reported to identify atelectatic areas corresponding to those detected on high-resolution CT [11]. One study reported 100% specificity for atelectasis detection by LUS [10].

Selected studies also described the use of LUS for monitoring physiotherapy or ventilatory interventions [9].

Pleural effusion and pleural abnormalities: Pleural effusion was reported infrequently, with prevalence generally ranging from 2.5% to 5.6%, and higher rates observed in cohorts evaluated during pulmonary exacerbations [9,10,13]. Where assessed, good agreement between LUS and CT was reported [8].

Pleural line abnormalities, including irregularity, fragmentation, blurring, and thickening, were reported more frequently and predominantly involved lower lung zones [8,12]. Jaworska et al. reported pleural abnormalities in a substantially higher proportion of cystic fibrosis patients compared with healthy controls [12].

Bronchiectasis and other findings: Bronchiectasis showed variable detectability by LUS. Early reports described low sensitivity of LUS for detecting bronchiectasis, particularly tubular or centrally located forms, compared with CT [8]. Later studies reported improved detection of saccular bronchiectasis, especially when associated with mucus plugging and peripheral involvement [11,15].

Diagnostic performance differed according to bronchiectasis morphology, with higher specificity reported for saccular forms and limited sensitivity for cylindrical bronchiectasis [11]. Findings related to air trapping or emphysema were rarely reported and inconsistently characterized [8,16].

LUS scoring systems in cystic fibrosis: Across the nine included studies, substantial heterogeneity was observed in the design, structure, and validation of LUS scoring systems. Four studies developed original cystic fibrosis-specific LUS scores [8,9,11,12], whereas the remaining studies either applied previously published scoring systems or performed structured descriptive ultrasound assessment without creating a new score [10,13-16]. Overall, the evolution of LUS scoring in cystic fibrosis reflects a progression from simple regional pattern-based approaches toward more complex multiparametric severity indices integrating structural and functional correlations.

Among the studies that developed original CF-specific scores, Strzelczuk-Judka et al. introduced one of the earliest structured systems, the Cystic Fibrosis Ultrasound Score (CF-USS), designed as a composite regional score with a maximum of 40 points [8]. The thorax was divided into four regions (anterior and posterior for each hemithorax), each evaluated across five domains, including pleural line abnormalities, focal and coalescent B-lines, subpleural consolidations, and pleural effusion. This score demonstrated a moderate but statistically significant correlation with the modified Chrispin-Norman chest radiograph score (Spearman R = 0.52, p = 0.0002), which strengthened to R = 0.81 in repeated examinations. Importantly, the authors highlighted the high sensitivity of ultrasound for detecting small subpleural consolidations, which were identified in approximately three-quarters of patients and frequently not visible on chest radiography [8].

Peixoto et al. subsequently proposed a more structured semi-quantitative CF-specific score based on the evaluation of 12 thoracic regions [9]. Each region was scored according to the presence of interstitial syndrome (B-pattern) and consolidation, with consolidation weighted more heavily, yielding a maximum score of 36. This system was among the first to demonstrate significant correlations with both structural and functional markers of disease severity. The LUS score correlated positively with the modified Bhalla CT score and negatively with spirometric indices, including forced expiratory volume in 1 second (FEV1), forced vital capacity (FVC), and forced expiratory flow between 25% and 75% of vital capacity (FEF25-75), supporting its potential role as a radiation-free surrogate marker of structural lung involvement. Agreement between ultrasound and CT severity was further supported by Bland-Altman analysis.

A more complex severity-weighted CF-specific scoring system was proposed by Ciuca et al., who incorporated a broader range of ultrasound findings, including multiple B-line patterns, subpleural consolidations of varying size, larger consolidations with or without bronchogram, and atelectasis across 12 lung regions [11]. Their score demonstrated one of the strongest structural validations reported to date, with a very strong correlation with the modified Bhalla CT score (Spearman rs = 0.87, p < 0.001). Significant correlations were also observed with lung clearance index (LCI) and spirometric parameters, indicating that ultrasound-detected peripheral structural abnormalities reflect functional impairment.

Jaworska et al. developed the most comprehensive CF-specific LUS score in terms of cohort size and feature integration [12]. In a large pediatric population, they proposed a composite score ranging from 0 to 44 that incorporated pleural abnormalities, B-line distribution, small and large consolidations, and CF-specific artefacts such as Am-lines. The score demonstrated strong correlations with radiographic severity assessed by the modified Chrispin-Norman score, as well as with pulmonary function indices, including FEV1, FVC, and LCI. Higher ultrasound scores were also observed in patients with chronic *Pseudomonas* *aeruginosa* infection and fungal colonization, suggesting potential sensitivity to microbiological disease burden. Interobserver agreement for both individual findings and total score was high, supporting reproducibility. A comparative overview of CF-specific LUS scoring systems is presented in Table 5.

**Table 5:** Summary of cystic fibrosis-specific lung ultrasound scoring systems Abbreviations: CF = cystic fibrosis; LUS = lung ultrasound; CXR = chest radiography; CT = computed tomography; FEV1 = forced expiratory volume in 1 second; FVC = forced vital capacity; FEF25–75 = forced expiratory flow between 25% and 75% of vital capacity; LCI = lung clearance index

Score (Author, Year)	Lung Regions Assessed	Maximum Score	Main Components	Validation Reference Standard	Key Reported Correlations
CF-USS (Strzelczuk-Judka et al., 2019) [8]	4 regions	40	Pleural line abnormalities; focal and coalescent B-lines; subpleural consolidations; pleural effusion	Modified Chrispin–Norman (CXR)	R = 0.52 (p = 0.0002); R = 0.81 in repeated examinations
Lung Ultrasound Score (Peixoto et al., 2020) [9]	12 regions	36	Interstitial syndrome (B-pattern); subpleural consolidations (weighted scoring)	Modified Bhalla (CT)	Positive correlation with CT severity; negative correlation with FEV1, FVC, and FEF25–75
LUS-CF Score (Ciuca et al., 2022) [11]	12 regions	36	B-line patterns (focal and coalescent); small and large subpleural consolidations; consolidations with bronchogram; atelectasis	Modified Bhalla (CT)	rs = 0.87 (p < 0.001); correlations with LCI and spirometric parameters
CF LUS Score (Jaworska et al., 2023) [12]	12 regions	44	Pleural line abnormalities; B-lines; Am-lines; small consolidations (≤10 mm); major consolidations (>10 mm); pleural effusion	Modified Chrispin–Norman (CXR)	Strong correlation with radiographic severity; correlations with FEV1, FVC, and LCI

Several studies did not develop new CF-specific scores but instead applied existing scoring systems. Curatola et al. used a previously published regional score derived from Peixoto et al., applying a 12-region 0-36 scoring framework during routine outpatient follow-up. In their cohort of clinically stable adolescents and young adults with cystic fibrosis, higher LUS scores correlated significantly with worse spirometric parameters, including lower FEV1, FVC, and FEF25-75, reinforcing the utility of this simple regional score for longitudinal monitoring [14].

Gräger et al. also used a Peixoto-derived scoring approach, but modified its application by extending scoring to all acquired ultrasound images rather than regional summaries. In this adapted method, each image was scored for B-lines and consolidations, resulting in a maximum possible score of 144 [16]. Although no new score was formally developed, this modification increased sensitivity for subtle abnormalities. The modified score demonstrated excellent discriminatory performance between cystic fibrosis patients and healthy controls (AUC = 0.968), although correlations with pulmonary function were not significant.

Marzook et al. did not employ a CF-specific scoring system but instead applied the Mongodi LUS aeration score, originally developed for general lung aeration assessment in critical care and not tailored to cystic fibrosis [17]. This six-zone score (0-36) was used to compare children with cystic fibrosis and primary ciliary dyskinesia. Correlation between the LUS score and chest radiography assessed by the modified Chrispin-Norman score was poor in the cystic fibrosis cohort (r ≈ 0.03), suggesting that generic aeration scores may not adequately capture CF-specific structural abnormalities [15].

Crispino et al. focused on asymptomatic preschool children with cystic fibrosis and did not apply a numerical LUS score. Instead, they used a structured point-of-care ultrasound protocol with 12-region scanning and descriptive recording of findings. Small subpleural consolidations (<1 cm) were significantly more frequent in cystic fibrosis compared with healthy controls (approximately 47% vs 2%) and were identified as the most relevant early ultrasound marker of subclinical disease. No correlation with CT, chest radiography, or pulmonary function was performed [13]. Similarly, Hassanzad et al. did not develop a composite score but evaluated specific LUS findings against CT during pulmonary exacerbations, demonstrating high diagnostic accuracy for consolidation and pleural abnormalities compared with HRCT [10].

**Figure 2:**
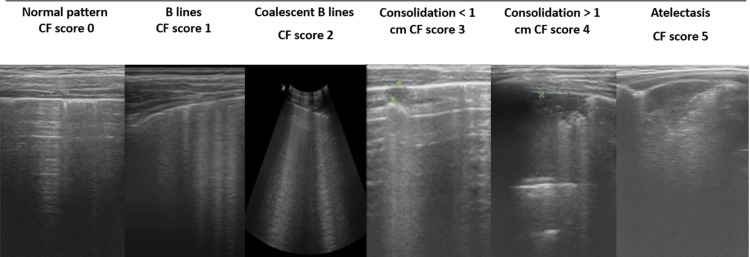
Cystic fibrosis-specific lung ultrasound severity scoring system Representative lung ultrasound images illustrating progressive pulmonary involvement in cystic fibrosis. Score 0 demonstrates a normal lung ultrasound pattern characterized by a smooth pleural line and horizontal A-lines. Scores 1–2 indicate increasing interstitial involvement with discrete and coalescent B-lines, respectively. Scores 3–4 show subpleural consolidations measuring <1 cm and >1 cm, respectively. Score 5 represents lung atelectasis. Adapted from Ciuca et al. [11], with permission from the publisher. Abbreviations: CF = cystic fibrosis.

A schematic representation of the cystic fibrosis-specific LUS scoring system, adapted from Ciuca et al., is presented in Figure 2.

Comparison of LUS With CT and CXR

Comparison of LUS findings or scores with conventional imaging varied substantially across studies. Three studies correlated ultrasound with CT [9-11]. Ciuca et al. demonstrated a very strong correlation between their LUS score and modified Bhalla CT score (rs = 0.87), while Peixoto et al. also reported a significant correlation between the LUS score and CT-based structural severity. Hassanzad et al., although not using a composite score, showed a high diagnostic accuracy of ultrasound for detecting consolidations and pleural abnormalities compared with CT, with an area under the curve of approximately 0.90 for consolidation detection.

Three studies compared ultrasound findings with chest radiography [8,12,15]. Strzelczuk-Judka et al. reported a moderate correlation between CF-USS and the modified Chrispin-Norman radiographic score (R = 0.52), and Jaworska et al. found strong associations between their composite LUS score and radiographic severity [8,12]. In contrast, Marzook et al. reported no meaningful correlation between the Mongodi LUS score and chest radiography in cystic fibrosis patients, likely reflecting both the use of a non-CF-specific scoring system and the mild disease burden in the cohort [15]. The remaining studies did not include a systematic comparison with CT or chest radiography.

Correlation of LUS Scores With Pulmonary Function and Microbiological Status

Several studies demonstrated significant associations between LUS scores and functional or microbiological markers of disease severity, particularly when CF-specific scoring systems were used.

Ciuca et al. reported strong correlations between their CF LUS score and lung clearance index (rs ≈ 0.80, p < 0.001), together with significant negative correlations with FEV1 and mid-expiratory flow parameters, indicating that higher ultrasound scores reflected worse ventilation inhomogeneity and airflow limitation [11]. Similar findings were reported by Peixoto et al., who observed significant negative correlations between LUS score and FEV1 (r ≈ −0.62, p < 0.01), FVC, and FEF25-75 [9]. Jaworska et al. confirmed these results in a larger cohort, demonstrating strong correlations between higher LUS scores and reduced spirometric indices and LCI, and additionally reporting significantly higher ultrasound scores in patients with chronic Pseudomonas aeruginosa infection and fungal colonization (p < 0.05) [12].

Curatola et al., using a Peixoto-derived score in clinically stable patients, also reported significant associations between higher LUS scores and lower FEV1, FVC, and FEF25-75 (all p < 0.05) [14]. In contrast, studies applying non-CF-specific scores or descriptive protocols showed weaker correlations. Gräger et al. and Marzook et al. did not observe significant relationships between the LUS score and pulmonary function [15,16], and Crispino et al. did not assess functional correlations [13].

Overall, correlations with pulmonary function and microbiological status were most consistent in studies using CF-specific LUS scoring systems, supporting their relevance for disease severity assessment and longitudinal monitoring [8,9,11,12].

Clinical Role of LUS in Cystic Fibrosis

LUS was most frequently investigated as a bedside, radiation-free imaging modality for evaluating pulmonary involvement in cystic fibrosis and for supporting longitudinal follow-up. Most studies, including those by Strzelczuk-Judka, Ciuca, Jaworska, Curatola, Marzook, Crispino, and Gräger, explored its utility in detecting peripheral structural lung abnormalities such as subpleural consolidations, pleural irregularities, and interstitial patterns in both clinically stable patients and individuals with more advanced disease [8,11-16].

The potential role of LUS in the assessment of pulmonary exacerbations was specifically examined by Hassanzad, Marzook, and partially by Peixoto, who reported a higher prevalence of consolidations, coalescent B-lines, and pleural abnormalities during exacerbation episodes compared with stable clinical conditions [9,10,15]. These observations suggest that LUS findings may reflect acute inflammatory changes and short-term fluctuations in disease activity.

Use of LUS as a monitoring tool was described in several studies, particularly those by Ciuca, Curatola, Peixoto, Jaworska, and Gräger, which included either longitudinal evaluation of clinically stable patients or short-term reassessment following therapeutic interventions [9,11-14,. In these cohorts, ultrasound findings were reported to vary in parallel with clinical status or disease severity markers, supporting the feasibility of repeated bedside assessment.

Across the included literature, LUS was consistently presented as a well-tolerated and repeatable technique, especially suitable for pediatric populations requiring serial imaging. However, authors such as Strzelczuk-Judka, Peixoto, and Ciuca emphasized that its clinical use should be considered complementary rather than substitutive to computed tomography, given the known limitations of ultrasound in detecting centrally located bronchiectasis and deeper parenchymal abnormalities [8,9,11].

Discussions

LUS is increasingly explored as a complementary imaging modality in cystic fibrosis, particularly in the context of improved survival and quality of life associated with multidisciplinary care and highly effective CFTR modulator therapies [11]. As the clinical course of cystic fibrosis evolves, imaging strategies are shifting from episodic assessment of advanced disease toward repeated monitoring of structural changes over time. In this setting, LUS offers a feasible, radiation-free tool for detecting and monitoring peripheral lung involvement.

The included studies consistently demonstrated that LUS can detect peripheral structural abnormalities, such as subpleural consolidations, pleural irregularities, and interstitial patterns, in patients with cystic fibrosis. Several authors reported that ultrasound identified lesions not visible on chest radiography, particularly small subpleural consolidations, suggesting increased sensitivity for peripheral disease [8,12]. Strong correlations between CF-specific LUS scores and CT structural severity were reported by Ciuca et al. and Peixoto et al. [9,11], while Jaworska et al. additionally demonstrated associations with pulmonary function and microbiological status [12]. These findings indicate that CF-adapted scoring systems may provide clinically meaningful information and reflect disease burden when structural abnormalities extend to the pleural surface.

The diagnostic performance of LUS appeared to vary according to disease severity. Studies including patients with moderate structural involvement reported stronger correlations between ultrasound scores and CT findings or functional parameters [11,12], whereas cohorts with predominantly mild disease showed weaker associations [15,16]. This pattern likely reflects the intrinsic limitation of ultrasound in visualizing central airway pathology, such as bronchiectasis and mucus plugging, when these changes do not reach the pleura. Similar observations have been emphasized in contemporary imaging reviews, which underline that CT remains the reference standard for comprehensive structural evaluation, while alternative modalities are needed to support repeated monitoring with lower radiation exposure [18].

The potential clinical value of LUS lies primarily in longitudinal assessment and follow-up. Its lack of ionizing radiation, bedside availability, and feasibility for repeated outpatient evaluation were highlighted across several studies [9-11]. Curatola et al. and Gräger et al. demonstrated the practicality of incorporating LUS into routine monitoring of clinically stable patients [14,16], while Ciuca et al. and Jaworska et al. showed that CF-specific ultrasound scores may track disease severity and functional impairment [11,12]. Early peripheral abnormalities detected in asymptomatic or mildly affected patients further suggest a possible role for identifying subclinical disease progression [13].

Compared with existing narrative literature, including the review by Boni et al. [6], the present study provides a structured synthesis of available evidence with particular focus on CF-specific ultrasound scoring systems and their validation against structural and functional outcomes. Studies applying CF-adapted scoring systems consistently showed stronger correlations with CT severity and pulmonary function than those using generic aeration scores, highlighting the importance of disease-specific approaches for meaningful interpretation. However, substantial heterogeneity persists in scanning protocols, scoring methods, and study populations, limiting direct comparison and emphasizing the need for standardization.

Overall, LUS cannot replace CT for a comprehensive assessment of cystic fibrosis lung disease, particularly for central airway pathology. Nevertheless, current evidence supports its role as a complementary, radiation-free imaging modality for detecting and monitoring peripheral lung abnormalities, particularly in pediatric and longitudinal follow-up settings.

Strengths and limitations of LUS in cystic fibrosis

LUS demonstrated consistent ability to detect peripheral structural abnormalities in cystic fibrosis, particularly subpleural consolidations, pleural irregularities, and interstitial patterns. Several studies reported that LUS identified lesions not visible on chest radiography, most notably small subpleural consolidations. This was highlighted by Strzelczuk-Judka and Jaworska, and further supported by Crispino, who detected subclinical peripheral abnormalities in asymptomatic preschool children. Ciuca and Peixoto showed strong correlations between CF-specific LUS scores and CT structural severity, while Jaworska also demonstrated associations with pulmonary function and microbiological status, supporting the clinical relevance of CF-adapted scoring systems.

Diagnostic performance appeared stronger in patients with more advanced structural involvement. Studies including patients with moderate disease severity, such as those by Ciuca, Peixoto, and Jaworska, reported strong correlations between LUS scores and CT findings or functional parameters [9,11,12]. In contrast, studies involving predominantly mild disease populations, including Gräger and Marzook, showed weaker correlations with pulmonary function or radiographic severity, suggesting lower sensitivity in early or minimally symptomatic disease. This observation may also reflect the relatively mild disease burden and the increasing use of CFTR modulator therapies in some cohorts, which may reduce the extent of peripheral structural abnormalities detectable by ultrasound.

The main strengths of LUS were its radiation-free nature, bedside availability, and feasibility for repeated outpatient monitoring, as emphasized by Curatola and Gräger. However, several limitations were consistently noted, including reduced sensitivity for central airway pathology such as bronchiectasis and mucus plugging, operator dependence, and substantial heterogeneity in scanning protocols and scoring systems across studies. In addition, the predominance of pediatric cohorts, with limited representation of adult patients, restricts the generalizability of findings to older CF populations. Air trapping and emphysematous changes were rarely and inconsistently characterized across studies, representing an important gap in current ultrasound-based assessment of cystic fibrosis lung disease. In addition, the relatively small number of studies and methodological heterogeneity in ultrasound protocols, scoring systems, and reference standards limited the possibility of quantitative meta-analysis.

Overall, current evidence suggests that LUS is particularly sensitive for detecting peripheral and more advanced structural lung disease in cystic fibrosis and may play a useful complementary role in monitoring disease progression, while its sensitivity for very early or central airway disease remains limited.

Based on current evidence, LUS may be most beneficial in specific clinical scenarios, including: (1) routine longitudinal monitoring in pediatric patients to reduce cumulative radiation exposure; (2) bedside assessment during suspected pulmonary exacerbations; (3) follow-up of known peripheral consolidations; and (4) adjunctive evaluation when spirometric changes occur without immediate access to CT. However, CT remains essential for the comprehensive assessment of central airway pathology.

## Conclusions

Lung ultrasound represents a promising complementary imaging modality in cystic fibrosis, capable of detecting peripheral structural abnormalities and reflecting disease severity when CF-specific scoring systems are used. Evidence from the included studies suggests that ultrasound correlates with structural and functional markers of disease, particularly in patients with established lung involvement, while offering the advantages of being radiation-free, accessible, and suitable for repeated monitoring. However, its limited sensitivity for central airway pathology and the heterogeneity of current protocols prevent its use as a standalone diagnostic tool. Standardization of scanning methods and CF-specific scoring systems, together with larger multicenter and longitudinal studies, are needed to better define the role of lung ultrasound within multimodal imaging strategies for cystic fibrosis.

## References

[1] Stoltz DA, Meyerholz DK, Welsh MJ (2015). Origins of cystic fibrosis lung disease. N Engl J Med.

[2] Wielpütz MO, Eichinger M, Biederer J (2016). Imaging of cystic fibrosis lung disease and clinical interpretation. Rofo.

[3] Wielpütz MO, Mall MA (2015). Imaging modalities in cystic fibrosis: emerging role of MRI. Curr Opin Pulm Med.

[4] Volpicelli G, Elbarbary M, Blaivas M (2012). International evidence-based recommendations for point-of-care lung ultrasound. Intensive Care Med.

[5] Pereda MA, Chavez MA, Hooper-Miele CC (2015). Lung ultrasound for the diagnosis of pneumonia in children: a meta-analysis. Pediatrics.

[6] Boni A, Cristiani L, Majo F (2024). Use of lung ultrasound in cystic fibrosis: is it a valuable tool?. Children (Basel).

[7] O'Connell OJ, McWilliams S, McGarrigle A (2012). Radiologic imaging in cystic fibrosis: cumulative effective dose and changing trends over 2 decades. Chest.

[8] Strzelczuk-Judka L, Wojsyk-Banaszak I, Zakrzewska A, Jończyk-Potoczna K (2019). Diagnostic value of chest ultrasound in children with cystic fibrosis - pilot study. PLoS One.

[9] Peixoto AO, Marson FA, Dertkigil SS (2020). The use of ultrasound as a tool to evaluate pulmonary disease in cystic fibrosis. Respir Care.

[10] Hassanzad M, Kiani A, Abedini A (2021). Lung ultrasound for the diagnosis of cystic fibrosis pulmonary exacerbation. BMC Pulm Med.

[11] Ciuca IM, Pop LL, Dediu M, Stoicescu ER, Marc MS, Manea AM, Manolescu DL (2022). Lung ultrasound in children with cystic fibrosis in comparison with chest computed tomography: a feasibility study. Diagnostics (Basel).

[12] Jaworska J, Buda N, Kwaśniewicz P, Komorowska-Piotrowska A, Sands D (2023). Lung ultrasound in the evaluation of lung disease severity in children with clinically stable cystic fibrosis: a prospective cross-sectional study. J Clin Med.

[13] Crispino AA, Musolino AM, Buonsenso D, Caloiero M, Concolino D (2024). Point of care lung ultrasound in preschool children with cystic fibrosis: a case-controlled, prospective, pilot study. J Ultrasound.

[14] Curatola A, Corona F, Squillaci D, Saccari A, Chiaretti A, Barbi E, Maschio M (2024). Lung ultrasound evaluation in people with cystic fibrosis: a new approach in the pulmonology outpatient clinic. Pediatr Pulmonol.

[15] Marzook N, Dubrovsky AS, Muchantef K, Zielinski D, Lands LC, Shapiro AJ (2024). Lung ultrasound in children with primary ciliary dyskinesia or cystic fibrosis. Pediatr Pulmonol.

[16] Gräger S, Puschmann M, Lorenz M, Krämer M, Mentzel HJ, Glutig K (2025). Lung ultrasound in children with cystic fibrosis - a new promising approach. Respir Med.

[17] Mongodi S, De Luca D, Colombo A (2021). Quantitative lung ultrasound: technical aspects and clinical applications. Anesthesiology.

[18] Ciet P, Bertolo S, Ros M (2022). State-of-the-art review of lung imaging in cystic fibrosis with recommendations for pulmonologists and radiologists from the "iMAging managEment of cySTic fibROsis" (MAESTRO) consortium. Eur Respir Rev.

